# Response of photomorphogenesis and photosynthetic properties of sweet pepper seedlings exposed to mixed red and blue light

**DOI:** 10.3389/fpls.2022.984051

**Published:** 2023-02-07

**Authors:** Yan Li, Guofeng Xin, Qinghua Shi, Fengjuan Yang, Min Wei

**Affiliations:** ^1^ College of Horticultural Science and Engineering, Shandong Agricultural University, Tai’an, Shandong, China; ^2^ Scientific Observing and Experimental Station of Environment Controlled Agricultural Engineering in Huang-Huai-Hai Region, Ministry of Agriculture, Tai’an, Shandong, China

**Keywords:** sweet pepper, light spectrum, growth, plant morphology, photosynthesis

## Abstract

Various light spectra, especially red (RL) and blue light (BL), have great effects on physiological processes and growth of plants. Previously, we revealed that the plant photomorphogenesis and photosynthesis of sweet pepper was significantly altered under BL or mixed RL and BL. The present study aimed to elucidate how mixed RL and BL influences plant photosynthesis during photomorphogenesis. We examined the growth, plant morphology, photosynthetic response of sweet pepper seedlings under monochromatic RL, BL, different ratios of mixed RL and BL (9R1B, 6R1B, 3R1B, 1R1B, 1R3B) with the same photosynthetic photon flux density of 300 μmol·m^-2^·s^-1^. White light (WL) were used as a control. The findings showed that the elongation of hypocotyl and first internode as well as leaf expansion were all stimulated by RL, while significantly restrained by BL compared with WL. Conversely, the leaf development, biomass accumulation and photosynthetic properties were inhibited by RL but promoted by BL. Additionally, compared with WL and other treatments, 3R1B could significantly improve the net photosynthetic rate, gas exchange, photosynthetic electron transport capacity, photochemical efficiency, shoot and root biomass accumulation. Furthermore, seedlings grew robustly and exhibited the greatest value of seedling index when exposed to this treatment. Overall, these results suggested that pepper seedlings grown under 3R1B performed better, possibly due to the more balanced light spectrum. It was more conducive to improve the plant photomorphogenesis and photosynthesis of sweet pepper, and a higher biomass accumulation and energy utilization efficiency could be achieved simultaneously under this mixed light spectrum.

## Introduction

Plants have different morphological and physiological responses to specific light spectra, and among the different light spectra, red (RL) and blue light (BL), which focus on more light absorption by chlorophyll than other wavelengths, are most efficiently utilized for the photosynthesis and phytochemical metabolism in plants (Han et al., 2017; Monostori et al., 2018). RL is generally regarded as the fundamental spectrum for plant growth and RL-absorbing phytochromes (phys) plays a key role in regulating leaf morphogenesis, photosynthetic apparatus formation and carbohydrate accumulation (Rehman et al., 2017). BL is recognized by photoreceptors such as cryptochromes (crys) and phototropins (phots), and these photoreceptors regulate chloroplast development, chlorophyll biosynthesis and stomata opening (Savvides et al., 2012).

However, monochromatic RL or BL could not satisfy the requirement of normal plant growth. Various studies have found that the mixed RL and BL was an effective lighting source to plant development and a suitable proportion of RL and BL accelerate photosynthesis and growth of sweet pepper and tomato (Li et al., 2017; Li et al., 2020). Therefore, the mixed RL and BL is used nowadays more and more in research and can be applied for the commercial cultivation of horticultural crops in controlled and semi-controlled environments (Miao et al., 2016).

Sweet pepper (*Capsicum annuum* L.) is one of economically important vegetables and widely cultivated in greenhouses worldwide. A prolonged period of RL and BL treatment has an apparent impact on growth and physiology of pepper seedlings (Tang et al., 2019). Moreover, in recent years, plant factories have developed rapidly, and light-emitting diodes (LEDs) as a kind of artificial light with the characteristics of high light efficiency, narrowly-centered spectrum and low energy consumption, have been frequently applied to manipulate the plant growth, development and metabolism in plant factories (Matsuda et al., 2016; Liu et al., 2018). In a previous study, we found that a suitable proportion of mixed RL and BL accelerated sweet pepper seedlings’ photosynthesis and growth (Li et al., 2020). However, the mechanism of how light spectra of mixed RL and BL regulate leaf photosynthesis and plant photomorphogenesis, as well as the optimal ratio of RL and BL, which could benefit pepper seedling growth remains unclear. Therefore, in this study, we investigated photosynthesis capacity, biomass accumulation and morphological acclimation of pepper seedlings under various proportions of mixed RL and BL.

## Materials and methods

### Plant material and climate conditions

The experiment was performed from May to September, 2016 in a Chinese solar greenhouse (China, 36°N, 117°E) in Shandong Agricultural University. The germinated sweet pepper (*Capsicum annuum* L. cv. HA-2502) seeds were sown in plastic trays with 50 holes (54 cm length × 30 cm width × 4.4 cm depth) filled with a mixture of peat and vermiculite (2:1, v/v). Three weeks later, when their second true leaf expanded fully, seedlings were transplanted into plastic pots (8 cm length × 8 cm width × 10 cm depth, one plant per pot). After that, 320 seedlings were selected and moved into an environmentally controlled growth chamber, where the average air temperature, relative humidity (RH), photoperiod and CO_2_ concentration were 26/18 °C (day/night), 70%, 12 h/12 h and 400 μmol·mol^-1^, respectively, and then, randomized into eight groups and were cultured under eight light spectra treatments for 30 d. From each light treatment, randomly five plants were sampled after periods of 6, 12, 18, 24 and 30 d after treatment (DAT) by LEDs. There were three replicates with a total of 40 seedlings for each treatment.

### Light treatments

**Figure 1:**
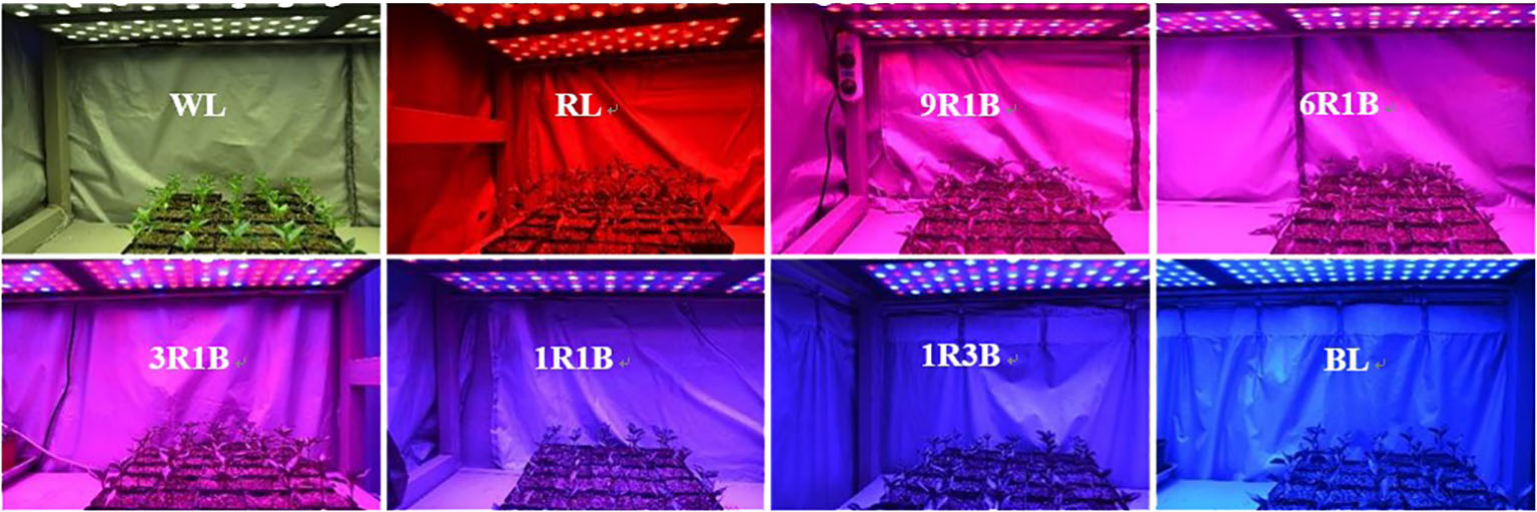
General view and spectral distribution of eight light spectra treatments.

All the mixed LEDs had the uniform spectra of RL and BL, and were designed by Chunying Optoelectronics Technology Co., Ltd., Guangdong, China. All treatments were performed in different layers of cultivation racks, which were covered by opaque silver plastic reflective cloth to prevent the light disturbance from the adjacent treatments (Li et al., 2021). Plants were subjected under different light conditions: monochromatic BL (peak intensity: 457 nm) and RL (peak intensity: 657 nm), mixed RL and BL (9:1, 9R1B; 6:1, 6R1B; 3:1, 3R1B; 1:1, 1R1B; 1:3, 1R3B: 90%, 85%, 75%, 50%, 25% RL at a wavelength of 657 nm and 10%, 15%, 25%, 50%, 75% BL at a wavelength of 457 nm, respectively), and a multiwavelength white light (WL, as control) with the same photosynthetic photon flux density (PPFD) of 300 μmol·m^-2^·s^-1^. PPFD was recorded using a light meter with a quantum sensor (LI-250 and LI-190R, Li-Cor Inc., Lincoln, NE, USA) and was 10 cm away from the top of seedling canopy to the bottom of LED lighting panels. The spectral photon flux density distributions (SPDs) of RL, BL and WL was measured using a spectroradiometer (Unispec-SC Spectral Analysis System, PP Systems Inc., Haverhill, MA, USA) (**Figure 1**).

### Measurements of plant morphological parameters

Plant height, first internode length and hypocotyl length were measured by a meter rule (cm), respectively, whereas the stem diameter was determined at the internode 1 cm above the cotyledona using a digital vernier caliper (mm, CD-20CPX, Mitutoya Corp., Kawasaki, Japan). The fully expanded true leaves and second fully expanded true leaves were collected to determine leaf number and leaf length, respectively. The seedlings including shoots and roots were dried to a constant weight at 75°C to measure the dry weight (DW). Before this measurement, the substrate particles attached to the roots were washed gently in distilled water. Root configuration indexes at 15 and 30 DAT, including total root length, root surface area, root volume, average diameter and tips number, were scanned with scanner (Epson Expression 10000G J181A, Japan) and leaf area (LA) of the fully developed young leaves at 15 and 30 DAT were monitored by a CI-202 leaf area measurer (CID Bio-Science Inc., Camas, WA, USA), and then, the data were analyzed with WinRHIZO (Model LA600, Regent Instruments Inc., Quebec, QC, Canada). The specific leaf area (SLA) and seedling index were determined using the following formulas:


$$\text{SLA}\left({\text{cm}}^{2}\cdot \text{m}{\text{g}}^{-1}\text{DW}\right)=\text{total LA }\left({\text{cm}}^{2}\right)/\text{leaf DW}\left(\text{mg}\right)$$

Seedling index=(Stem diameter(mm)/Plant height (cm)+Root DW(g)/Shoot DW (g))

×DW (g)

### Photosynthetic pigment concentration measurement

On 15 and 30 DAT, samples (0.5 g) from the fresh second fully expanded leaves were collected and incubated in 25 mL of 95% (v/v) ethanol reagent in darkness for 24-36 h at room temperature until the leaves became completely colorless. Afterwards, absorbance of the supernatant at 663, 646 and 470 nm were recorded by a spectrophotometer (UV-2450, Shimadzu Corp., Japan), respectively, and the concentration of chlorophyll *a* (Chl *a*), *b* (Chl *b*) and total carotenoid (Car) were measured based on the methods described by Sartory and Grobbelaar (1984).

### Gas exchange parameters measurement

Gas exchange parameters including net photosynthetic rate (*P*n), stomatal conductance (*G*s), intercellular CO_2_ concentration (*C*i), transpiration rate (*T*r) and stomatal limitation (*L*s) of the second functional leaf of pepper seedlings were determined by a LI-6400 gas exchange analyzer (Li-Cor Inc., Lincoln, NE, USA) at 30 DAT. The conditions in the assimilation chamber of the LI-6400XT equipment such as PPFD with 90% RL and 10% BL, RH, CO_2_ concentration, leaf temperature and flow rate were 300 µmol·m^-2^·s^-1^, 70%, 400 µmol·mol^-1^, 25°C and 400 mL·min^-1^, respectively.

### Chlorophyll a fluorescence parameters measurement

Chl *a* fluorescence was investigated on same leaf and position mentioned above at 30 DAT using a FMS-2 chlorophyll fluorometer (Hansatech Instruments Ltd., King Lynn, Norfolk, UK). To standardize the measuring conditions and ensure that all of the photosynthesis system II (PSII) reaction centers were open when the maximal photochemical quenching was determined, seedlings were dark-adapted for 20 min prior to the evaluations, and then, minimum fluorescence (*F*_o_) and maximum fluorescence (*F*_m_) were recorded. Chl a fluorescence parameters, maximal quantum yield of PSII (*F*_v_/*F*_m_), effective quantum yield of PSII (*Φ*_PSII_), electron transport rates (ETR) and photochemical quenching (*qP*) were calculated according to Li et al. (2021).

### Root vigor measurement

Root vigor was analyzed by the triphenyl tetrazolium chloride (TTC, Sinopharm Chemical Reagent Co., Ltd., Shanghai, China) method (Wang et al., 2006). In brief, 0.5 g fresh root was immersed in 10 mL of equally mixed solution of 0.4% TTC and phosphate buffer and kept in the dark at 37°C for 2 h. Subsequently, 2 mL of 1 mol/L H_2_SO_4_ was added to stop the reaction with the root. The root was dried with filter paper and then extracted with ethyl acetate. The red extractant was transferred into the volumetric flask to reach 10 mL by adding ethyl acetate. The absorbance of the extract at 485 nm was read. Root vigor was expressed as TTC reduction intensity and TTC reduction was calculated using the following formula:


Root vigor=amount of TTC reduction (µg)/fresh root weight (g)time (h)

### Statistical analysis

All values were presented as the mean ± standard errors (SE) with three replicates each. Data were analyzed by one-way analysis of variance (ANOVA) using SPSS 16.0 (SPSS Institute Inc., Cary, NC, USA), and the differences between the means were tested using Duncan’s multiple range test at *P*< 0.05 level. The graphs were performed by Origin 8.5 (OriginLab Institute Inc., Northampton, MA, USA). 

## Results

### Plant morphology and biomass accumulation

**Figure 2:**
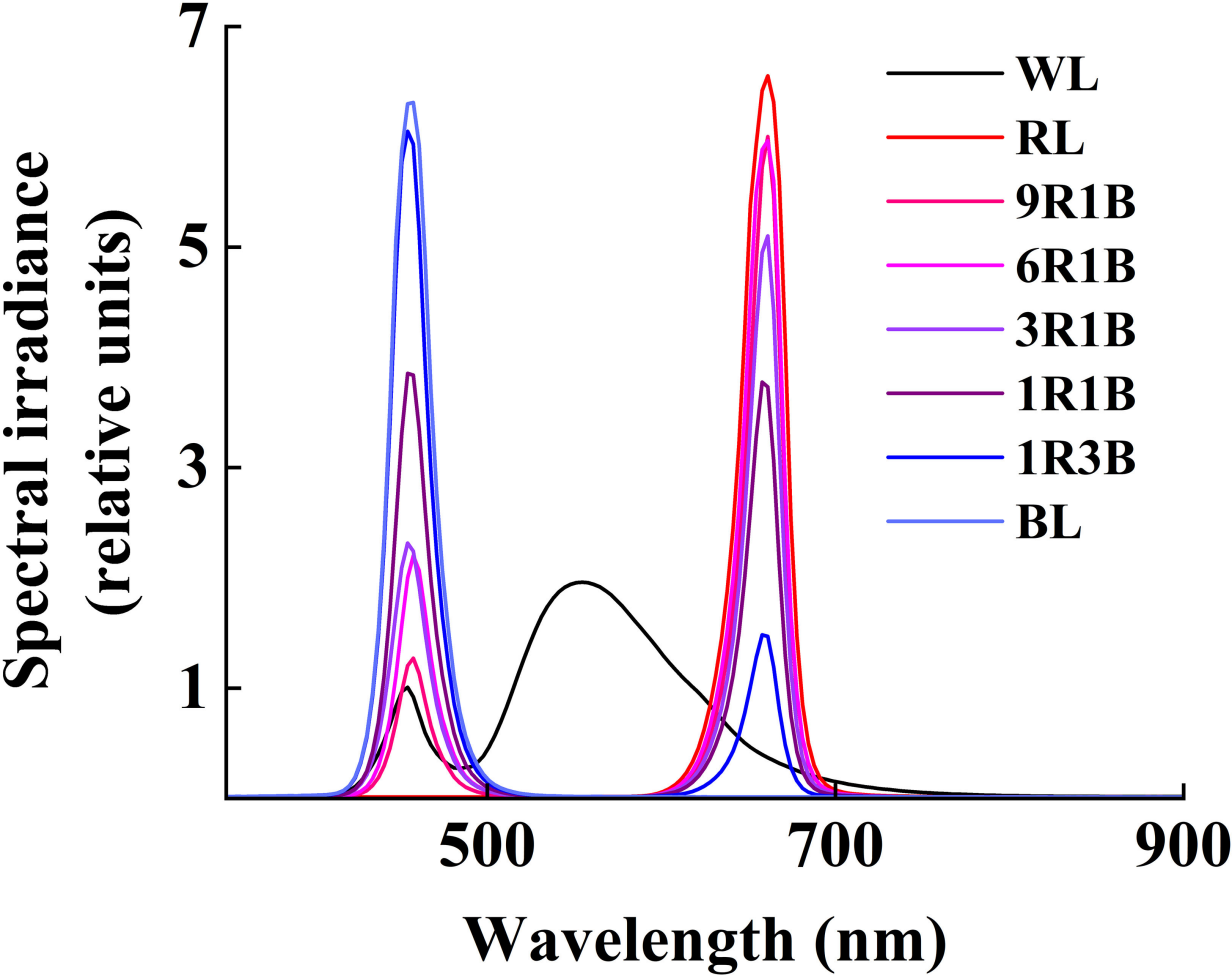
Effect of monochromatic and mixed red and blue light on plant morphology of sweet pepper seedlings at 30 day after treatment.

**Figure 3:**
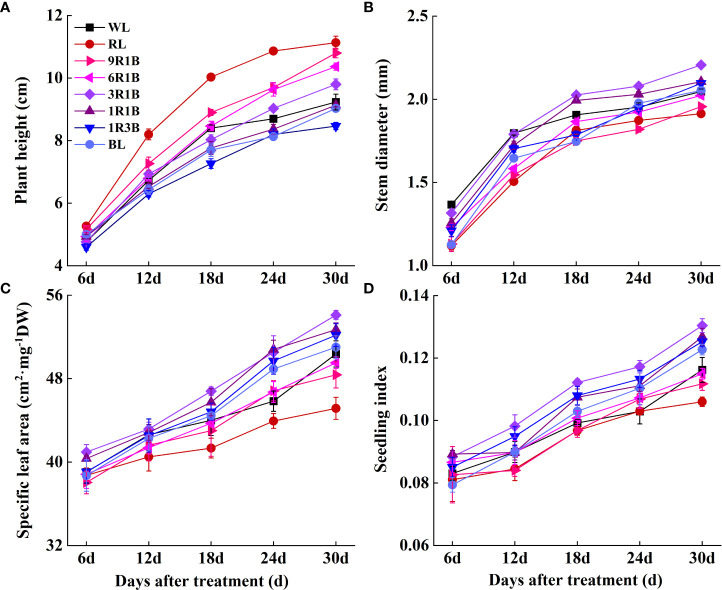
Effect of monochromatic and mixed red and blue light on growth of sweet pepper seedlings throughout 30 days after treatment. **(A)** plant height; **(B)** stem diameter; **(C)** specific leaf area; **(D)** seedling index.

**Figure 4:**
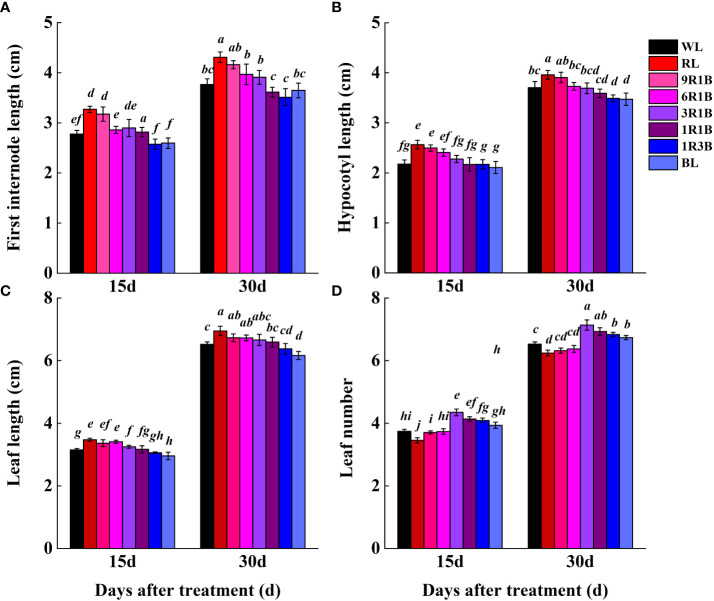
Effect of monochromatic and mixed red and blue light on plant morphology of sweet pepper seedlings at day 15 and 30 after treatment. **(A)** first internode length; **(B)** hypocotyl length; **(C)** leaf length; **(D)** leaf number. Difference in lower-case letters indicates significant difference at *P* < 0.05.

**Figure 5:**
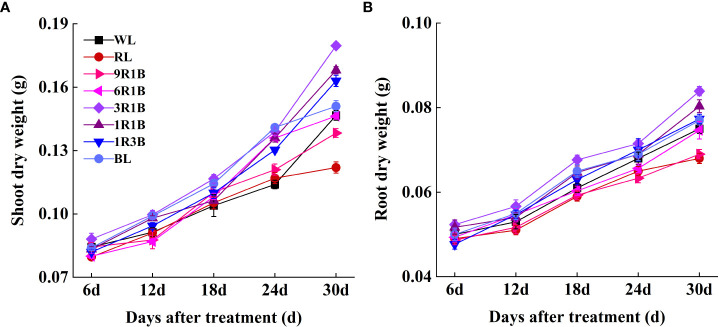
Effect of monochromatic and mixed red and blue light on **(A)** shoot dry weight and **(B)** root dry weight of sweet pepper seedlings throughout 30 days after treatment.

As shown in **Figure 2**, the morphology of sweet pepper seedlings at 30 DAT was found to be significantly affected by the monochromic and mixed RL and BL. Compared to WL, plant height grown under 1R3B was significantly reduced (*P*< 0.05), followed by plants grown under BL and 1R1B at 30 DAT, while RL produced the tallest plants (**Figure 3A**). The length for first internode, hypocotyl and leaf of seedlings under all light spectra exhibited a similar trajectory (**Figures 4A–C**). Relative to WL, the stem diameter was significantly greater for plants treated by 3R1B (*P*< 0.05), but thinner for plants under other treatments, although there were no statistically significant differences under WL and BL (*P* > 0.05) during the last experimental period and the smallest for plants under RL (**Figure 3B**). Moreover, seedlings under 3R1B had higher values for SLA, seedling index, leaf number, shoot and root DW (**Figure 3C, D**; **Figure 4D**; **Figures 5A, B**). The least values for these parameters were recorded in the RL-treated seedlings, respectively.

Plants under 3R1B had higher values for total root length, root surface area, root volume, average diameter and tips number comparing with WL and other treatments at 15 and 30 DAT (**Table 1**). The least values for all these parameters were recorded in the 1R3B-treated plants. Moreover, the total root length and root volume were not statistically different between 1R3B- and RL-illuminated plants at 30 DAT (*P* > 0.05), but these were significantly lower than WL (*P*< 0.05). Similarly, the total root length, root surface area, root volume and tips number of 6R1B- and 9R1B-treated seedlings were statistically the same.

**Table 1:** Effect of monochromatic and mixed red and blue light on root configuration indexes of sweet pepper seedlings at day 15 and 30 after treatment.

Treatment period (d)	Treatments	Total root length (cm)	Root surface area (cm^2^)	Root volume (cm^3^)	Average diameter (mm)	Tips number
15d	WL	118 ± 5.11 b	10.67 ± 0.52 bc	0.203 ± 0.02 b	0.234 ± 0.003 b	219 ± 5.22 b
	RL	72 ± 3.28 cd	5.57 ± 0.41 d	0.097 ± 0.01 d	0.195 ± 0.003 d	141 ± 4.31 d
	9R1B	115 ± 4.37 b	10.57 ± 0.27 bc	0.180 ± 0.01 bc	0.218 ± 0.002 c	210 ± 6.37 b
	6R1B	120 ± 6.12 b	10.90 ± 0.54 b	0.197 ± 0.01 bc	0.221 ± 0.003 c	217 ± 2.78 b
	3R1B	173 ± 5.45 a	13.67 ± 0.52 a	0.273 ± 0.02 a	0.250 ± 0.003 a	253 ± 4.29 a
	1R1B	111 ± 4.33 b	10.07 ± 0.38 c	0.173 ± 0.02 c	0.218 ± 0.004 c	212 ± 5.37 b
	1R3B	65 ± 4.21 d	5.50 ± 0.44 d	0.097 ± 0.02 d	0.184 ± 0.002 e	135 ± 4.03 d
	BL	77 ± 4.09 c	5.87 ± 0.43 d	0.110 ± 0.01 d	0.187 ± 0.002 e	153 ± 6.28 c
30d	WL	358 ± 4.12 b	36.07 ± 0.62 b	0.703 ± 0.01 b	0.408 ± 0.003 b	495 ± 9.16 b
	RL	176 ± 6.23 e	18.53 ± 0.63 e	0.357 ± 0.01 e	0.339 ± 0.003 e	260 ± 5.33 f
	9R1B	232 ± 6.34 d	30.37 ± 0.53 c	0.420 ± 0.01 d	0.362 ± 0.005 c	409 ± 6.32 c
	6R1B	241 ± 4.78 cd	29.67 ± 0.72 c	0.420 ± 0.01 d	0.348 ± 0.005 d	399 ± 4.24 c
	3R1B	457 ± 12.56 a	42.90 ± 0.67 a	0.900 ± 0.01 a	0.442 ± 0.004 a	672 ± 8.17 a
	1R1B	355 ± 6.33 b	30.83 ± 0.92 c	0.630 ± 0.02 c	0.347 ± 0.004 d	377 ± 3.45 d
	1R3B	168 ± 4.29 e	15.90 ± 0.44 f	0.350 ± 0.02 e	0.305 ± 0.001 f	248 ± 6.12 g
	BL	252 ± 6.18 c	25.57 ± 0.91 d	0.430 ± 0.04 d	0.299 ± 0.007 f	297 ± 5.21 e

Data are presented as means ± SE, n = 5. Different letters indicate significant differences between values (P < 0.05). WL, white light; RL, monochromatic red light; BL, monochromatic blue light; 9R1B, mixed RL and BL of 9:1; 6R1B, mixed RL and BL of 6:1; 3R1B, mixed RL and BL of 3:1; 1R1B, mixed RL and BL of 1:1; 1R3B, mixed RL and BL of 1:3.

### Photosynthetic pigment content

**Figure 6:**
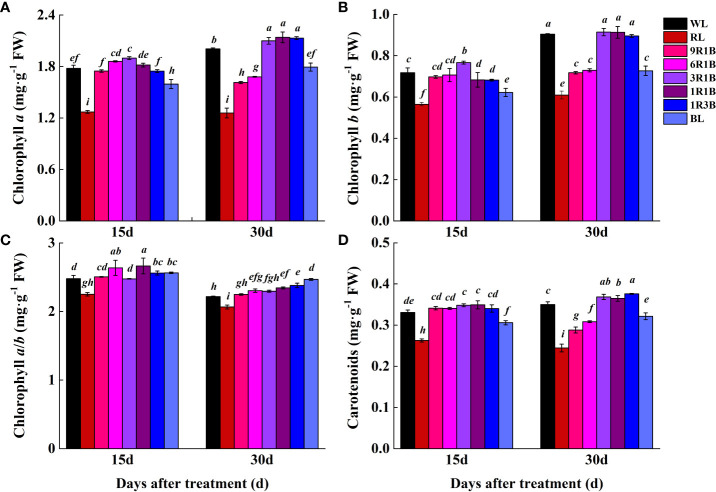
Effect of monochromatic and mixed red and blue light on photosynthetic pigment content in leaves of sweet pepper seedlings at day 15 and 30 after treatment. **(A)** chlorophyll *a*; **(B)** chlorophyll *b*; **(C)** chlorophyll *a*/*b*; **(D)** carotenoids. Difference in lower-case letters indicates significant difference at *P* < 0.05.

To examine the effects of mixed RL and BL on the photosynthetic pigment content, sweet pepper seedlings were exposed to different light spectra for 30 d (**Figure 6**) and the findings showed that significantly lower levels of Chl *a*, Chl *b* and Car were observed under RL, 9R1B, 6R1B, 1R3B and BL compared to WL at 30 DAT (*P*< 0.05), and the least values were observed in plants with the treatment of RL at 15 and 30 DAT. Higher levels of Chl and Car were detected in other treatments at each treatment period, while no significant difference was present among WL and them. BL yielded the highest Chl *a*/*b* at 30 DAT, followed by 1R3B, and the lowest Chl *a*/*b* was found for RL.

### Gas exchange and chlorophyll a fluorescence parameters

Generally, compared to WL, most leaf gas exchange parameters including *P*n, *G*s and *T*r were significantly increased in seedlings under 3R1B (*P*< 0.05, **Table 2**), at 14%, 28% and 29% higher than in WL, respectively, while these parameters were largely decreased by RL and were not statistically different between the WL and BL treatments (*P* > 0.05). Furthermore, the levels of *C*i and *L*s were found to follow the opposite tendency.

**Table 2:** Effect of monochromatic and mixed red and blue light on gas exchange parameters in sweet pepper seedlings at day 30 after treatment.

Treatments	*P*n (µmol·m^-2^·s^-1^)	*G*s (mmol·m^-2^·s^-1^)	*C*i (µmol·mol^-1^)	*T*r (mmol·m^-2^·s^-1^)	*L*s
WL	9.37 ± 0.22 b	0.25 ± 0.03 b	330.33 ± 15.21 c	2.34 ± 0.11 e	0.16 ± 0.01 c
RL	5.95 ± 0.31 e	0.17 ± 0.01 d	375.67 ± 12.83 a	1.73 ± 0.13 g	0.19 ± 0.01 a
9R1B	6.96 ± 0.20 d	0.19 ± 0.04 cd	343.67 ± 13.05 b	2.69 ± 0.08 d	0.16 ± 0.01 c
6R1B	7.57 ± 0.35 c	0.20 ± 0.01 c	325.02 ± 7.64 cd	2.78 ± 0.14 c	0.17 ± 0.01 b
3R1B	10.70 ± 0.23 a	0.32 ± 0.03 a	316.67 ± 14.91 e	3.01 ± 0.21 a	0.16 ± 0.01 b
1R1B	7.71 ± 0.56 c	0.26 ± 0.04 b	321.00 ± 10.54 d	2.86 ± 0.12 b	0.16 ± 0.01 c
1R3B	6.20 ± 0.19 e	0.18 ± 0.02 cd	336.67 ± 11.24 c	2.06 ± 0.09 f	0.17 ± 0.02 bc
BL	9.77 ± 0.34 b	0.29 ± 0.02 ab	318.33 ± 17.62 de	2.37 ± 0.07 e	0.15 ± 0.02 c

Data are presented as means ± SE, n = 5. Different letters indicate significant differences between values (P < 0.05). WL, white light; RL, monochromatic red light; BL, monochromatic blue light; 9R1B, mixed RL and BL of 9:1; 6R1B, mixed RL and BL of 6:1; 3R1B, mixed RL and BL of 3:1; 1R1B, mixed RL and BL of 1:1; 1R3B, mixed RL and BL of 1:3.

**Figure 7:**
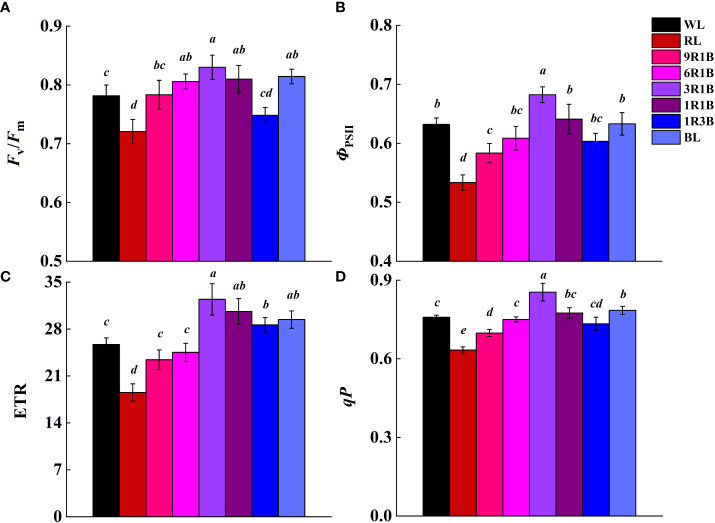
Effect of monochromatic and mixed red and blue light on chlorophyll *a* fluorescence parameters of leaves from sweet pepper seedlings at day 30 after treatment. **(A)** *F*_v_/*F*_m_; **(B)** *Φ*_PSII_; **(C)** ETR; **(D)** *qP*. Difference in lower-case letters indicates significant difference at *P* < 0.05.

The levels of *F*_v_/*F*_m_, *Φ*_PSII_, ETR and *qP* showed the similar trajectory that they were evidently enhanced by 3R1B relative to WL, except the values of *F*_v_/*F*_m_ and ETR was not statistically different among 6R1B, 1R1B, BL, and 1R1B, respectively (**Figure 7**). However, these parameters dropped drastically in RL-treated seedlings (*P<* 0.05). These results indicated that BL could raise the proportion of the reaction centers in PSII opening under light adaptation, enhance PSII reaction center activity, and improve the electron transfer rate.

### Root vigor

**Figure 8:**
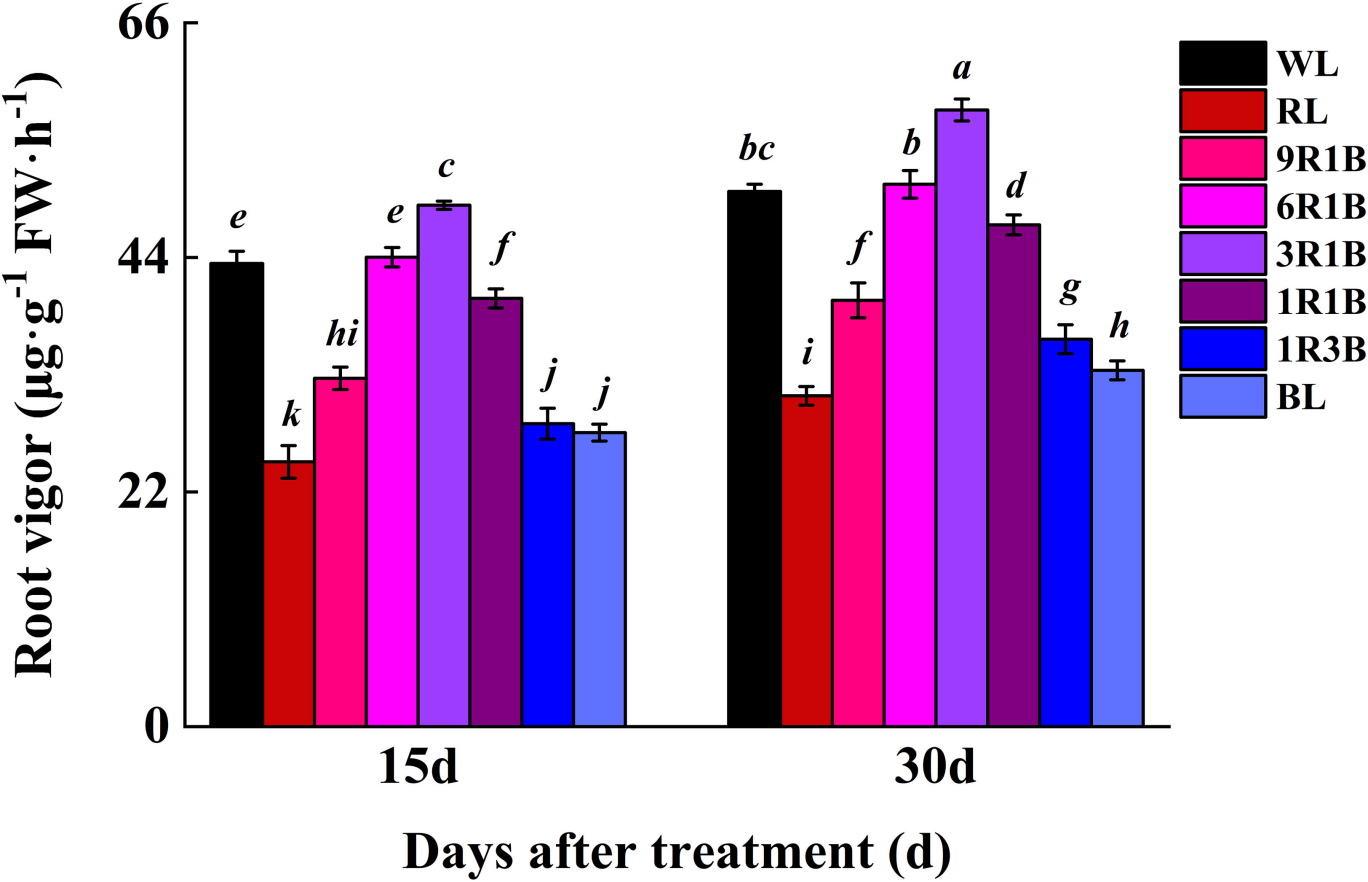
Effect of monochromatic and mixed red and blue light on root vigor of sweet pepper seedlings at day 15 and 30 after treatment. Difference in lower-case letters indicates significant difference at *P* < 0.05.

The root vigor was significantly higher in 3R1B-treated seedlings than that in WL (*P*< 0.05) and at 13% and 15% higher than that under WL at 15 and 30 DAT, respectively (**Figure 8**). However, the root vigor did not differ significantly between WL and 6R1B (*P* > 0.05), and this parameter was evidently lower in seedlings under other light treatments than in WL and the lowest level was observed in plants under RL.

## Discussion

For light-controlled development, it is generally assumed that the photoreceptors perceive and interpret incident light and transduce the signals to modulate light-responsive nuclear genes, amongst the spectral wavelengths, RL and BL are the primary spectral wavelengths and highly influence the plant photosynthesis, physiological metabolism and photomorphogenesis (Ooi et al., 2016). In the present research, the morphological and photosynthetic characteristics of sweet pepper seedlings were significantly influenced by light spectra. The results indicated that seedlings grown under mixed RL and BL, especially 3R1B, had improved plant growth parameters. This was consistent with sweet pepper seedlings in our previous study (Li et al., 2020).

In this study, we found that seedlings grown under RL revealed typical shade-avoidance syndrome, namely significantly enhanced plant height, internodal and hypocotyl length and this agreed with previous reports (Liu et al., 2011). It could be due to RL could stimulate hypocotyl and stem elongation (Izzo et al., 2020). Previous reports indicated that phyB is a major photoreceptor that mediates hypocotyl elongation inhibition under continuous RL (Shinomura et al., 1996). Therefore, RL may accelerate petiole and internode elongation by inactivating phyB, and then, inducing stem elongation by improved gibberellin (GA) levels, which affected by indole-3-acetic acid (IAA) (Li et al., 2021; Xiao et al., 2022). It has been reported that GA, brassinosteroid and auxin are involved in BL signaling through cry1 as the primary photoreceptor in the inhibition of hypocotyl elongation (He et al., 2019; Xu et al., 2021). However, unlike previous studies, the shortest seedlings were found under treatment of 1R3B in our present research. It is assumed that mixed RL and BL with higher ratio of BL were more inhibitive of hypocotyl and leaf elongation and inducing in the sweet pepper seedlings a more compact morphology with shorter stems and smaller leaf areas than monochromatic BL. These results indicated that increasing BL could inhibit cell division and expansion (Kang et al., 2021). Furthermore, lower levels of GA and IAA under a low RL/BL ratio of 1R3B may lead to reduced elongation of pepper seedlings’ shoots (Islam et al., 2014; Matsuo et al., 2019).

Biomass is an important indicator in determining seedling qualities. In this present study, the DW and seedling index of seedlings under 3R1B was greater than that of WL and other treatments, which may implicate that this spectrum applied in the experiment is optimized effectively, since it promotes development and drives photosynthesis as a result of the increased contents of chl *a* and total chlorophyll in seedlings (Tang et al., 2019). It was also reported that the mixed RL and BL might promote fresh weight and DW in chrysanthemum and sweet pepper (Liu et al., 2011; Li et al., 2020). The biomass of pepper shoots was significantly increased under 3R1B compared with WL and other treatments probably due to the enlarged SLA in this study. The larger leaf is a good indicator of higher photosynthetic surface area per unit investment in leaf tissue, which may have led to the significant increment in biomass (Dieleman et al., 2019). This was in agreement with Bugbee (2016) who concluded that the fraction of light intercepted by a crop is more closely related to biomass than the short-term effects on quantum efficiency of photosynthesis. Besides, mixed RL and BL could also increase shoot regeneration and development by stimulating cell division (Kwon et al., 2015).

Chlorophyll content is an important determinant of photosynthesis and dry matter accumulation. In addition, carotenoids play a vital role in photosynthesis by absorbing light and protecting chlorophyll from photo-oxidation (Carvalho et al., 2013; Tuan et al., 2013). Our present results showed that BL plays an important role in the synthesis of plant chlorophyll, and plants grown under RL showed the lowest levels of chlorophyll and carotenoids. This was consistent with previous studies (Dutta-Gupta and Jatothu, 2013; Anuchai and Hsieh, 2017). It indicated that BL was more essential than RL for normal photosynthesis by mediating photosystems activity and photosynthetic electron transport capacity (Miao et al., 2016). However, these were unlike to the results of Bianchetti et al. (2018) and Tian et al. (2019). This suggested that plant responded to RL and BL are species-specific (Liu et al., 2011; Cope et al., 2014). Different plant species, or the same species at different growth stages, show diverse responses to the same light spectra and display complexity and instability of photobiological reactions. Moreover, stimulation of photosynthetic pigments under 3R1B was consistent with our previous results obtained in tomato seedlings (Li et al., 2017; Li et al., 2021). It might be due to the synergistic effect of RL and BL when the supplement of appropriate proportion for BL in RL has the property of promoting the biosynthesis of chlorophyll, and this property may be further reinforced when they work together (Mao and Guo, 2018).

Photosynthetic rate is a key factor affecting plant assimilation capacity and yield. A lower *P*n in plants under monochromatic RL has been shown in several crops (Hogewoning et al., 2010; Li et al., 2017; Li et al., 2021). Our data were consistent with previous results. This may be attributed to the excess ROS suppressed repair of photodamaged PSII and increased the degree of photodamage, which directly induced relatively low rate of CO_2_ assimilation and redundant *C*i under RL (Takahashi and Murata, 2008). In addition, the beginning of the leaf senescence is accompanied by the lower *P*n and the degradation of related proteins (Hensel et al., 1993). Hence, RL significantly reduced *P*n and *F*_v_/*F*_m_, which might result in acceleration of leaf senescence (Su et al., 2014). The photodamaged PSII induced by RL could be alleviated by BL, which could benefit pepper seedling growth by improving light use efficiency and diminish photoinhibition (Miao et al., 2019; Kang et al., 2021). On the other hand, the *P*n and *F*_v_/*F*_m_, *Φ*_PSII_, ETR and *qP* of seedlings exposed to 3R1B treatment was found to be significantly higher than those of other light spectra. These were consistent with our previous results (Li et al., 2017; Li et al., 2021), which indicated that the photosynthetic efficiency in pepper seedlings was promoted under this treatment, thereby more electrons were absorbed, captured and transported. Apparently, the increased concentration of light absorbing pigments in leaves has considerable consequences for leaf CO_2_ uptake under this treatment. This may be also related to the well-developed stomata (Li et al., 2020; Kang et al., 2021), higher stomatal conductance (Hamedalla et al., 2022) and high nitrogen accumulation in leaves under this spectral wavelength (Fan et al., 2022). Consequently, the morphology of pepper seedlings under this light spectrum revealed higher SLA, FW and DW of shoots and roots, and thus better performance compared to other treatments.

Vigorous roots support shoot growth by fully supplying the plant with water and mineral nutrition. Moreover, root tip is an important site where root absorb water and nutrient in plants. The more root tips number, the longer root length, and the more root surface are, the more beneficial to capture nutrient effectively (Zhang et al., 1999). In this study, the growth and morphological features of 3R1B-treated pepper seedlings exhibited a tight appearance, but the shoot and root structure of RL- or 1R3B-treated seedlings were adversely affected plant performance, indicating poor root growth under RL and 1R3B. Therefore, growth and development are dependent on the spectral wavelength of light, and the addition of RL may have further increased plant growth and development since stronger RL might better penetrate the plant canopy than weaker RL. Perhaps 3R1B treatment achieved a balanced spectral environment by supplementing a favorable amount of RL to pepper seedlings at 300 μmol·m^-2^·s^-1^ PPFD (Lin et al., 2021). This agreed with our previous results obtained in tomato seedlings (Li et al., 2017). According to Manivannan et al. (2015), this growth may be related to the ability of 3R1B treatment to induce the formation of endogenous GA, which is an important growth regulator involved in cell elongation. Furthermore, the increase in root length and formation of adventitious roots for pepper seedlings under 3R1B may be due to IAA levels, as this treatment promoted polar IAA transport from apical shoot to downward portion, which regulated by genes involved in auxin formation and signal transduction as well as auxin transportation (Liu et al., 2011; Shen et al., 2022), and thus longer root under 3R1B in this study might be related to higher IAA accumulation in root. Therefore, the better root development of pepper seedlings exposed to 3R1B leads to faster acclimatization.

## Conclusion

This research provided a better understanding of the responses of growth and photosynthesis in sweet pepper seedlings exposed to mixed RL and BL. The mixed RL and BL had significant effects on the photosynthesis, morphogenesis and growth of sweet pepper seedlings. It was found that proper proportion for RL and BL of 3R1B promoted plant growth by controlling and optimizing photosynthetic performance. This was mainly manifested by an improvement in the *P*n, *F*_v_/*F*_m_, *Φ*_PSII_, ETR, *qP*, increasing SLA and photosynthetic pigment content. Furthermore, this might ensure healthy chloroplast development under this treatment to achieve a higher photosynthetic capacity, thereby improving the growth and biomass accumulation of seedlings. These findings suggested that 3R1B was the most effective light treatment among spectral wavelength in this study for producing high quality of sweet pepper seedlings.

## Data availability statement

The original contributions presented in the study are included in the article/supplementary material. Further inquiries can be directed to the corresponding authors.

## Author contributions

YL and MW conceived the original research plan and designed the study. GX performed experiments and analyzed the data. YL analyzed data and drafted the manuscript. All authors read and agreed to the final draft.
